# Evaluation of bacterial biofilm, smear layer, and debris removal efficacy of a hydro-dynamic cavitation system with physiological saline using a new ex vivo model: a CLSM and SEM study

**DOI:** 10.1186/s12903-025-05463-y

**Published:** 2025-01-18

**Authors:** He Liu, Xi Wang, Zhejun Wang, Ya Shen

**Affiliations:** 1 Division of Endodontics, Department of Oral Biological and Medical Sciences, Faculty of Dentistry, University of British Columbia, 2199 Wesbrook Mall, Vancouver, British Columbia V6T 1Z3 Canada; 2 Department of Anesthesiology, Pharmacology, and Therapeutics, Faculty of Medicine, University of British Columbia, Vancouver, British Columbia V6T 1Z3 Canada

**Keywords:** Biofilm, Confocal laser scanning microscopy, Debris, Odne^TM^Clean, Physiological saline, Scanning electron microscopy, Smear layer

## Abstract

**Introduction:**

To evaluate the bacterial biofilm, smear layer and debris removal efficacy of a hydro-dynamic cavitation system with physiological saline using a new ex vivo model.

**Methods:**

Seventy-five dentin discs were prepared from fifty-four extracted teeth. Seventy-five artificial root sockets were prepared. Sixty dentin discs were used to grow 3-week-old bacterial biofilms, while smear layer and debris were produced on fifteen dentin discs. These dentin discs were adhered to the middle third of the artificial root canals. The sixty ex vivo models with biofilm-covered dentin discs were divided into six groups: control, needle with physiological saline, ultrasonic with physiological saline, Odne™ Clean with physiological saline, needle with 3% NaOCl, and ultrasonic with 3% NaOCl. Biofilm removal efficacy was evaluated using confocal laser scanning microscopy. The fifteen ex vivo models with smear layer and debris-covered dentin discs were divided into three groups: control, Odne™ Clean with physiological saline, and 5% NaOCl followed by 17% EDTA. Smear layer and debris removal efficacy was evaluated using scanning electron microscopy. Statistical analysis was performed using one-way analysis of variance for comparisons involving more than two groups. Post-hoc pairwise comparisons were conducted using the Tukey test.

**Results:**

Odne^TM^Clean with physiological saline (98%) performed significantly better than needle irrigation (47%) or ultrasonic activation (54%) with physiological saline (*P* < 0.05). Odne^TM^Clean with physiological saline removed biofilms as effectively as needle irrigation (97%) or ultrasonic activation (98%) with 3% NaOCl (*P* > 0.05). Additionally, 5% NaOCl followed by 17% EDTA (score: 1.33) removed the smear layer significantly better than Odne^TM^Clean with physiological saline (score: 4.47) (*P* < 0.05). However, Odne^TM^Clean with physiological saline (score: 1.27) removed debris as effectively as 5% NaOCl followed by 17% EDTA (score: 1.13) (*P* > 0.05).

**Conclusions:**

Odne^TM^Clean with physiological saline can effectively remove bacterial biofilm and debris from the dentin surface but cannot effectively remove the smear layer. Utilizing Odne^TM^Clean during the final irrigation may enhance root canal cleaning efficacy.

**Supplementary Information:**

The online version contains supplementary material available at 10.1186/s12903-025-05463-y.

## Introduction

Root canal biofilms are highly structured communities of bacterial cells embedded in a hydrated extracellular polymeric substances (EPS) matrix and adherent to the surface of the root canal wall [1, 2]. The biofilm on the dentin surface is difficult to detach primarily due to the complex adhesion mechanisms between the biofilm and the dentin [3, 4]. The multilayer structure of the biofilm and the viscoelasticity of its EPS provide it with strong mechanical stability, allowing it to resist external shear forces and the flow of irrigating solutions [3, 4].

The smear layer is a thin layer of debris that forms on the surface of the root canal walls during instrumentation [5, 6]. It consists of organic and inorganic material, including dentin particles, pulp tissue remnants, and microorganisms. Dentin debris are small particles of dentin generated during the mechanical instrumentation of the root canal [7]. Both the smear layer and dentin debris can be penetrated by bacteria, potentially offering protection to biofilms adhering to the root canal wall [6–8]. Additionally, they can interfere with the tight adaptation of root canal sealers to the dentinal walls, promoting microleakage [9–11]. Removing the smear layer and dentin debris enhances dentin disinfection, reduces the potential for microleakage, and improves sealer penetration into the dentinal tubules [9–11].

During the last four decades, numerous irrigation techniques or systems have been proposed or designed to activate irrigants such as sodium hypochlorite (NaOCl) and Ethylenediaminetetraacetic acid (EDTA) to improve their removal efficacy in remaining biofilm, smear layer, and debris, or to facilitate their distribution and penetration into complex and intricate root canal configurations [12–17]. Physiological saline solution (0.85% NaCl) is biocompatible, non-toxic, and isotonic, minimizing tissue irritation, allergic reactions, and harmful residues [12, 15, 18]. However, it is not one of the main irrigants as it has neither tissue-dissolving nor antimicrobial activity [12, 15]. It is often used between two irrigating solutions, such as NaOCl and EDTA or chlorhexidine, to prevent chemical reactions between them [15]. The activation of physiological saline solution between two irrigating solutions during the final irrigation may help to remove remaining pulp tissue, biofilm, and debris left after root canal instrumentation [12, 15].

Recently, Odne^TM^Clean (Odne AG, Dübendorf, Switzerland), a hydro-dynamic cavitation-based irrigation system, has been developed for cleaning root canals during the final irrigation phase after root canal instrumentation. Unlike other irrigation technologies Odne^TM^Clean is claimed by the manufacturer to be the first to leverage physiological saline solution to generate a cavitation cloud of imploding microbubbles. These microbubbles produce shockwaves that propagate along the root canal, effectively removing residual pulp tissue, biofilm, smear layer, and debris from the canal walls. Compared to conventional irrigation systems, the use of physiological saline offers potential advantages, such as reduced toxicity, biocompatibility, and minimized chemical residues, while the cavitation mechanism enhances cleaning efficacy without relying on aggressive chemical agents. Furthermore, the system’s 190-micron plastic tip is highly flexible, conical, and designed to facilitate debridement following minimally invasive preparations (requiring a minimum canal size of 20.04). Despite these promising features, no published study has yet evaluated the efficacy of Odne^TM^Clean with physiological saline in removing bacterial biofilm, smear layer, and debris from the dentin surface.

Confocal laser scanning microscopy (CLSM) is a non-invasive imaging technique that not only provides qualitative images of biofilms but also allows for quantitative analysis [19, 20]. Therefore, CLSM with fluorescent dyes has been widely used to evaluate the effect of various irrigation techniques or irrigants on root canal biofilms [19, 20]. Scanning electron microscopy (SEM) is a valuable tool for evaluating the removal of the smear layer and debris, as it provides detailed, high-resolution images of the root canal surfaces and allows for quantitative analysis, such as measuring the amount of smear layer or debris using specific scoring criteria [21, 22].

The aim of this study was to compare the bacterial biofilm, smear layer and debris removal efficacy of the Odne^TM^Clean system with double side-vented needle irrigation and ultrasonic activation using a new artificial ex vivo model. Biofilm removal efficacy was evacuated using CLSM, while smear layer and debris removal efficacy were assessed using SEM. The null hypothesis was that there would be no significant differences in biofilm, smear layer or debris removal efficacy between the Odne^TM^Clean system and double side-vented needle irrigation or ultrasonic activation.

## Materials and methods

### Ethics approval

The clinical Research Ethics committee of the University of British Columbia (certificate H1202430) approved the protocol of this study. All participants provided signed informed consent, authorizing the use of their extracted teeth for research purposes. These teeth were extracted for reasons unrelated to the study. The authors ensure that all procedures were conducted in accordance with relevant guidelines and regulations.

### Selection of teeth

**Fig. 1:**
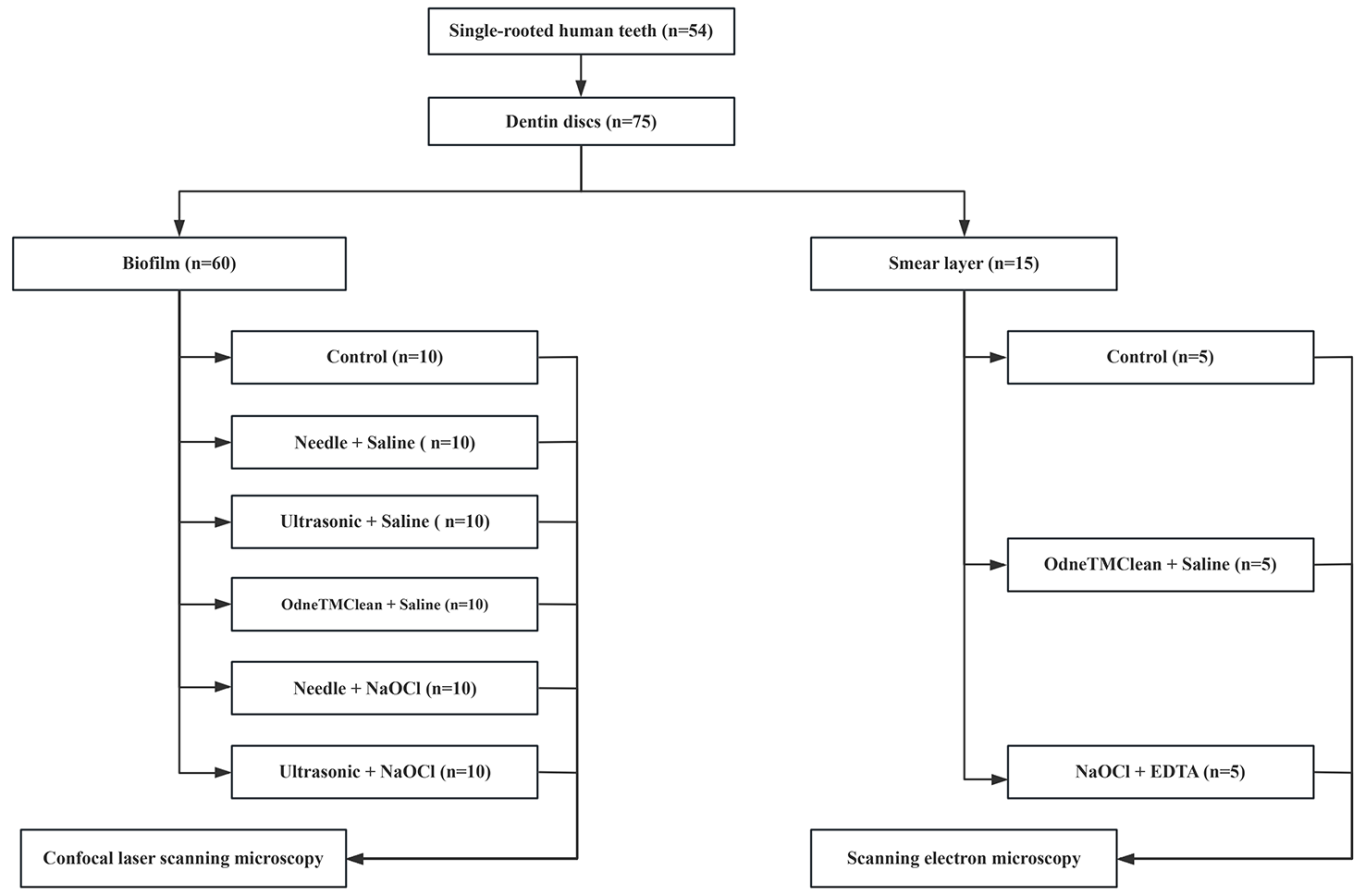
The flow chart of experiments in this study

**Fig. 2:**
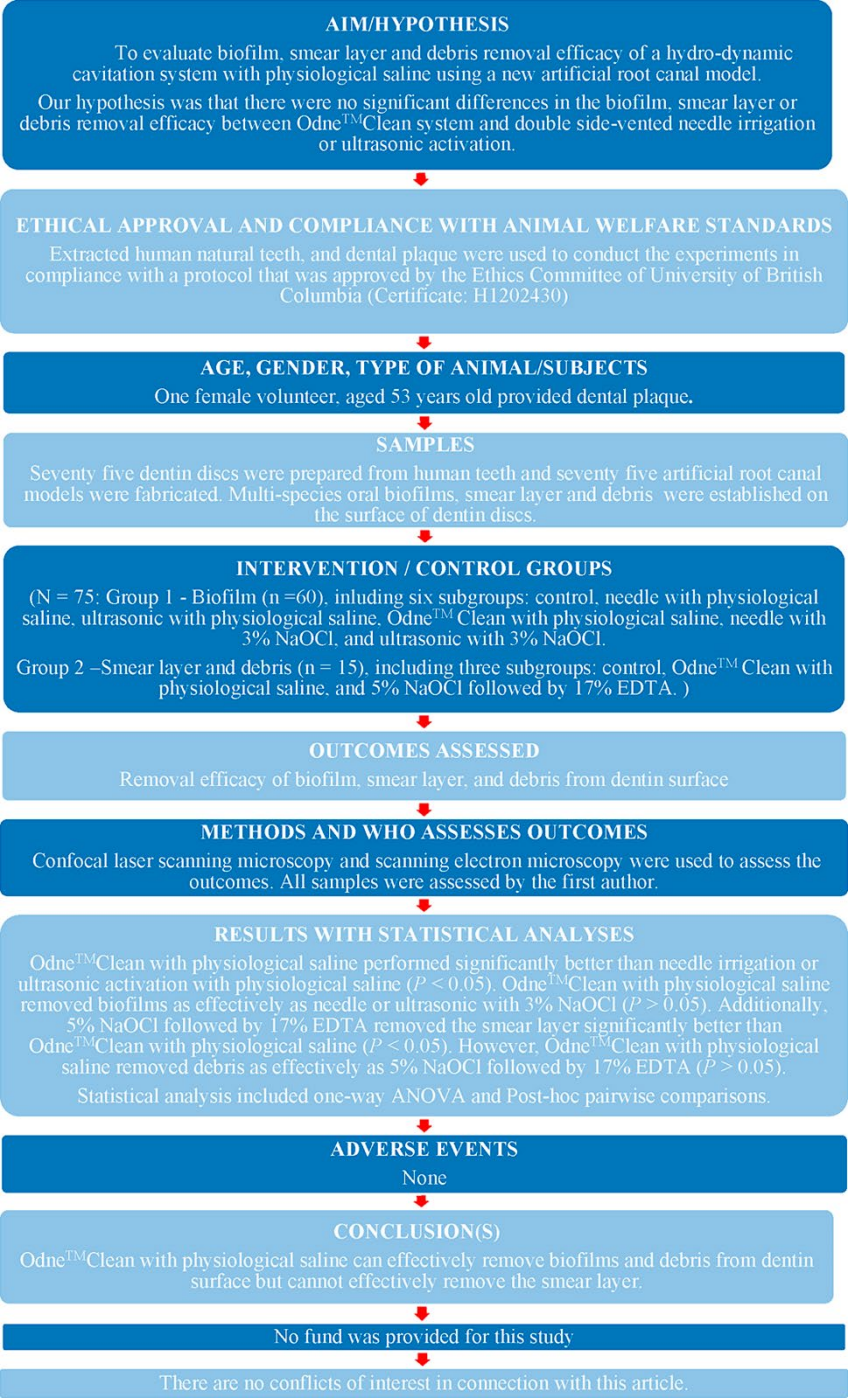
PRILE 2021 flow chart

Human permanent single-rooted teeth, extracted for reasons unrelated to the study, were obtained. Each tooth underwent visual and radiographic examination in both mesiodistal and buccolingual directions (Planmeca Intra, Helsinki, Finland). The radiographs were then analyzed using digital radiography imaging software (Planmeca Romexis, Helsinki, Finland). The inclusion and exclusion criteria are summarized in Table 1. A total of 54 single-root human permanent teeth were finally included in this study. The teeth were collected and stored in a 0.01% NaOCl solution at 4 °C prior to use. Figure 1 presents the flow chart of experiments in this study. This manuscript adheres to the Preferred Reporting Items for Laboratory Studies in Endodontology (PRILE) guidelines (Fig. 2) and follows the corresponding checklist [23].

**Table 1:** The inclusion and exclusion criteria of teeth samples

Inclusion criteria	Exclusion criteria
• Extracted teeth.	• Previous root canal treatment.
• Single-rooted teeth.	• Extensive caries or restorations reaching the root canal wall.
• Fully developed root apices.	• Incomplete root formation.
	• Visible cracks.
	• Internal or external resorption.
	• Severely calcified.

### Sample size calculation

Sample size calculation was performed using G*Power version 3.1 software (Heinrich-Heine-Universität Düsseldorf, Düsseldorf, Germany), with α = 0.05 and β = 0.80, and effect size f = 0.50 [24]. The test family (F tests) and statistical test (ANOVA: Fixed effects, omnibus, one-way) were selected. An a priori power analysis was conducted to compute the required sample size given the α level, power, and effect size. The biofilm removal efficacy, considered as the most important outcome in this study, was chosen to calculate the sample size. The parameters for the sample size calculation are presented in the supplementary document (Figure S1). The minimum number of samples calculated for each group was 10. Therefore, at least 10 dentin discs were required for biofilm culture in each group.

### Dentin discs preparation

Dentin discs used in this study were prepared from root dentin blocks using a modified method from previous studies [20, 25]. A root dentin block with a length of 4 mm was horizontally sectioned from each tooth using a diamond saw at 1000 rpm under water cooling. The root canals inside the blocks were enlarged to a size of Gates Glidden drill #6 (Tulsa Dentsply, Tulsa, OK, USA) at 300 rpm under water cooling.

Each cylindrical dentin block was split into two semicylindrical halves by first creating a thin groove in the middle with a low-speed handpiece and small round bur, followed by splitting with a blade and hammer. The outer surfaces of these halves were then ground with 600-grit silicon carbide paper to achieve a uniform thickness of 1 mm and to remove the root surface cementum. Subsequently, the dentin specimens were shaped into circular discs, measuring 3 mm in diameter and 1 mm in thickness, using a water-cooled low-speed handpiece with a fine carbide bur operating at 300 rpm. The smear layer on both sides of the dentin discs was removed by immersion in 5.25% NaOCl and 6% citric acid (Sigma-Aldrich, St Louis, MO, USA) each for 4 min. Dentin discs were irrigated with 5% Na_2_S_2_O_3_ (Sigma-Aldrich) to deactivate any remaining NaOCl [22]. Finally, 75 dentin discs were prepared.

### Biofilm culture

Sixty dentin discs were utilized as substrates for bacterial biofilms growth, using methods outlined in previous studies [26, 27]. These discs were coated overnight at 4 °C with bovine dermal type I collagen (Cohesion, Palo Alto, CA, USA) in each well of a 24-well plate. Written informed consent was obtained for plaque bacteria collection. Supragingival plaque was gathered from the first or second molars of an adult volunteer with healthy gingiva using sterile wooden tooth sticks and suspended in brain-heart infusion (BHI) broth (Becton Dickinson, Sparks, MD, USA). The bacterial suspension was adjusted to an optical density (OD) value of 0.08–0.10 (405 nm).

The collagen-coated dentin discs were rinsed in phosphate-buffered saline (PBS) (Sigma-Aldrich, St Louis, MO, USA) and subsequently immersed in 1.8 mL BHI broth and 0.2 mL of the dispersed dental plaque solution in a 24-well plate. The setup was incubated under anaerobic conditions (AnaeroGen Compact; Oxoid, Thermo Scientific, Dardilly, France) at 37 °C for three weeks, with fresh BHI broth refreshed once every week.

### Artificial ex vivo model

**Fig. 3:**
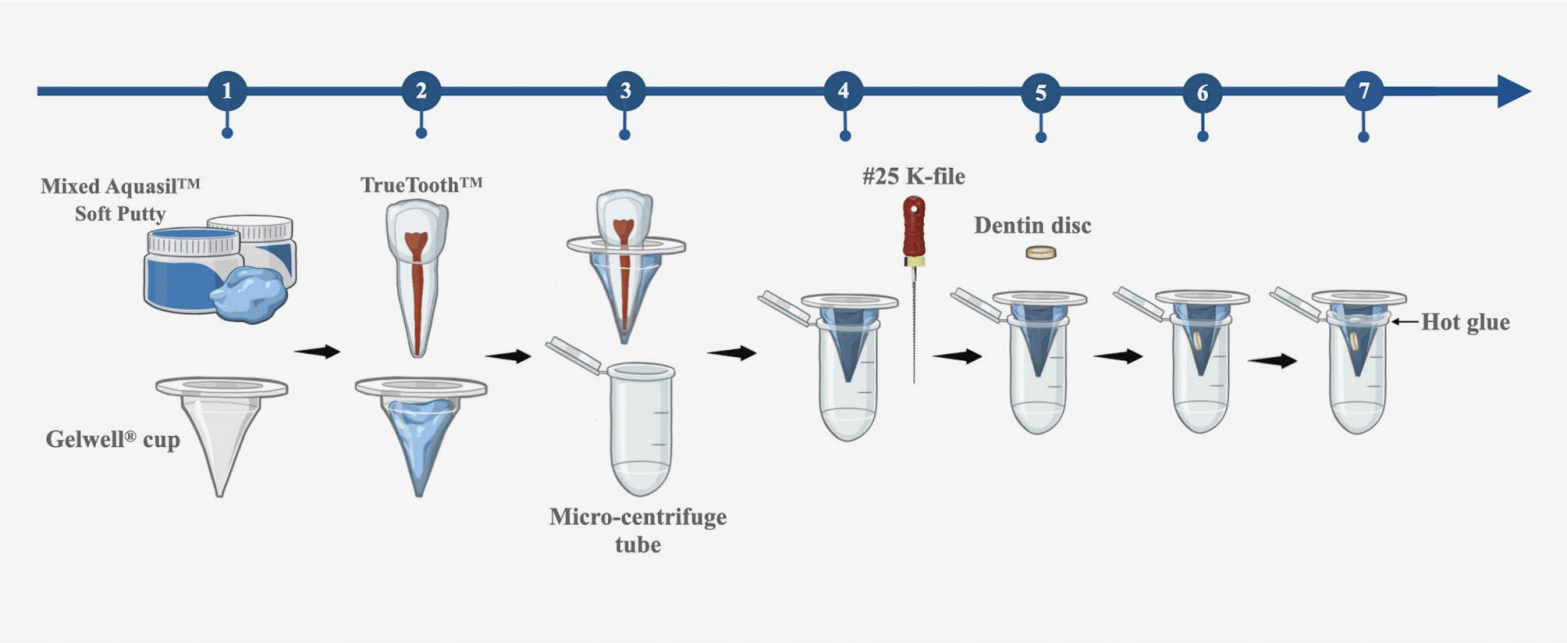
Schematic illustration presenting the preparation of artificial ex vivo model

Seventy-five artificial models were prepared using TrueTooth™ three-dimensionally (3D) printed maxillary incisor teeth (PlanB Dental, Santa Barbara, CA, USA), Aquasil™ Soft Putty (Dentsply Sirona, Konstanz, Germany), micro-centrifuge tubes (2.0 mL; Fisher Scientific, Pittsburgh, PA, USA), and Gelwell^®^ cups (Jordco, Beaverton, OR, USA). Figure 3 illustrates the process of preparing the artificial models.

Equal amounts of base and catalyst (ratio 1:1) were scooped out from their respective containers and mixed thoroughly until a uniform color was achieved. The mixed Aquasil™ Soft Putty was quickly placed into the Gelwell^®^ cup. The root of the 3D printed tooth was inserted into the mixed putty with pressure until the root tip met firm resistance. This pressure was maintained until the putty set. The root of the 3D printed tooth was then removed from the set putty. A #25 K-file was inserted into the artificial root socket until its tip was visible at the bottom of the Gelwell^®^ cup, successfully establishing the artificial model. This model was then placed into a micro-centrifuge tube, and the gap between the artificial model and the tube was sealed with hot glue (Acme Office Products, Orangeville, Canada).

### Biofilm removal

Endodontic irrigation devices and instruments used for biofilm removal included the 31-gauge double-side-vented irrigation needle (NaviTip™; Ultradent, South Jordan, UT, USA), the EndoUltra ultrasonic handpiece and NiTi activator tip (size 20, 0.02 taper; Vista Dental Products, Racine, WI, USA), and Odne™ Clean system. The ultrasonic irrigation needle and double-side-vented irrigation needle were connected to a 60 mL syringe filled with physiological saline (Sigma-Aldrich), 3% and 5% NaOCl solutions through a Luer lock connection to Tygon ST tubing (Ismatec, WertheimMondfeld, Germany). The 3% and 5% NaOCl solutions were prepared by diluting 6% NaOCl (Vista Dental Products) with distilled water. A digitally controlled peristaltic pump (Reglo Digital MS-2/8, Ismatec) was used to deliver the irrigant from the needle at a precise flow rate. Before each biofilm removal, the peristaltic pump and needle flow rates were calibrated as previously described [28].

A biofilm-covered dentin disc was inserted into the artificial root using pliers. The side of the dentin disc without the biofilm was adhered to the root canal wall with super glue, positioned 5 mm below the root canal orifice. Sixty artificial root canals with biofilm-covered dentin discs were divided into the following six groups (*n* = 10 each) according to the irrigation methods:

1. **Control group**: No irrigation.
2. **Needle + Physiological Saline group**: A 31-gauge double-side-vented irrigation needle (5mL/min) was used to irrigate the artificial canal with physiological saline for 30 s, 3 mm short from the working length (WL). The needle was moved in an up-and-down motion.
3. **Ultrasonic + Physiological Saline group**: A 31-gauge double-side-vented irrigation needle was used to fill the canal with physiological saline. A non-cutting EndoUltra NiTi activator tip, mounted on an ultrasonic handpiece, was inserted into the artificial canal 3 mm short from the WL and then activated for 30 s in an up-and-down motion without touching the root canal wall.
4. **Odne**^**™**^**Clean + Physiological Saline group**: The tip of Odne^™^ Clean was inserted into the artificial canal 3 mm short from the WL and then activated for 30 s in an up-and-down motion without touching the root canal wall.
5. **Needle + NaOCl group**: A 31-gauge double-side-vented irrigation needle (5mL/min) was used to irrigate the artificial canal with 3% NaOCl for 30 s, 3 mm short from the WL. The needle was moved in an up-and-down motion.
6. **Ultrasonic + NaOCl group**: A 31-gauge double-side-vented irrigation needle was used to fill the canal with 3% NaOCl. A non-cutting EndoUltra NiTi activator tip (size 20, 0.02 taper) mounted on an ultrasonic handpiece was inserted into the artificial canal 3 mm short from the WL and then activated for 30 s in an up-and-down motion without touching the root canal wall.

### Confocal laser scanning microscopy analysis

Dentin discs were carefully removed from the artificial root model to avoid touching the root canal walls of the discs. The dentine discs were then analyzed with CLSM as described previously [26, 27, 29]. The discs were stained with fluorescent LIVE/DEAD BacLight Bacterial Viability stain (Molecular Probes, Eugene, OR, USA) containing SYTO 9 (green) and propidium iodide (red), following the manufacturer’s instructions, and then rinsed with phosphate-buffered saline for 1 min. Fluorescence from each stained cell was visualized using a CLSM (FV10i-LIV, Olympus, Richmond Hill, Canada) at a 1024 × 1024-pixel resolution with a 10× objective lens. The excitation/emission wavelengths were 480/500 nm for SYTO 9 and 490/635 nm for propidium iodide. Biofilm on each dentin disk was scanned, capturing a stack of 100 slices at 0.5 μm intervals from the top to the bottom of the biofilm. Thresholds for the red and green fluorescence were manually set by adjusting the gain sensitivity in phase contrast mode to just below the point of overexposure. This ensured that overexposure spots disappeared without compromising fluorescence detection. The absolute threshold values were adjusted for different samples to prevent over- or underexposure [29]. The map view of each sample and the original confocal data was saved.

Confocal images were quantified and analyzed using Imaris 7.2 software (Bitplane Inc., St. Paul, MN, USA). The original confocal data was uploaded to Imaris 7.2 software, which automatically captured the intensity of green and red fluorescence across the full thickness of the biofilm layers. The software then reconstructed the 2-dimensional fluorescence intensity of all layers into a 3-dimensional volume stack [29]. Three-dimensional volume stacks were constructed, and the volume of bacterial fluorescence in each group was measured. The proportion of biofilm removal was calculated using the following equation: proportion of biofilm removal = (1 - bacterial fluorescence volume in each group / average bacterial fluorescence volume in control group) ×100%.

### Smear layer and debris production and removal

According to a previously described protocol [6], a smear layer was produced on the root canal walls of 15 dentin blocks using a medium-grit round bur for 4 s at 1500 rpm. A smear layer and debris-covered dentin disc was inserted into the artificial root canal using pliers. The side of the dentin disc without the smear layer and debris was adhered to the root canal wall with super glue, positioned 5 mm below the root canal orifice.

The fifteen dentin blocks covered with smear layer were randomly divided into three groups (*n* = 5 each):

1. **Control group**: No irrigation.
2. **Odne™ Clean group**: The root canal walls of the dentin discs were irrigated with Odne™ Clean and physiological saline in a 30-second cycle, repeated 10 times.
3. **5% NaOCl + 17% EDTA group**: The root canal walls of the dentin discs were treated with 5% NaOCl for 4 min, followed by 17% EDTA for 1 min.

### Scanning electron microscopy

The sample preparation was carried out according to a previously described protocol [21]. The dentin discs were subjected to serial dehydration using increasing concentrations of ethanol (50%, 70%, 80%, and 100%). The dehydrated specimens were then sputter-coated with iridium using a Leica EM MED020 Coating System (Leica Microsystems Inc, Concord, Canada). The surface of the dentin discs after treatment was imaged using an SEM (Helios Nanolab 650; FEI, Eindhoven, Netherlands).

Three random fields were selected and imaged from each dentin disc, resulting in a total of 45 images. These images were analyzed by two examiners who were blinded to the group distribution and were not involved in sample preparation, irrigation, or SEM image acquisition. The smear layer and debris on the dentin disc surfaces were evaluated and scored based on criteria from a previous study (Table 2) [22]. One examiner assessed the smear layer in the SEM images, while the other evaluated the debris. To ensure the reproducibility of the scoring system and consistency of intraobserver performance, the SEM images were reevaluated by the same examiner under identical conditions four weeks later. If discrepancies arose between the two scores of the same SEM image, the image was flagged, and the other examiner independently re-evaluated it. Any differences in the scores between the two examiners were resolved through discussion until a consensus was reached.

**Table 2:** Scores assigned to smear layer and debris evaluation

Score	Smear layer	Debris
1	No smear layer, with more than 90% of dentinal tubules open.	Clean root canal wall with only a few small debris particles.
2	Small amount of smear layer, with more than 50% of dentinal tubules open.	Few small agglomerations of debris, covering less than 25% of the surface.
3	Homogeneous smear layer covering the root canal wall, with only a few dentinal tubules open.	Many agglomerations of debris, covering less than 50% of the root canal wall.
4	Complete root canal wall covered by a homogeneous smear layer, with less than 25% of dentinal tubules open.	More than 50% of the root canal wall covered with debris.
5	Heavy, nonhomogeneous smear layer covering the entire root canal wall, with no open dentinal tubules.	Complete or nearly complete root canal wall covered by debris, more then 75%.

### Statistical analysis

Statistical analysis was conducted using GraphPad Prism software, version 10.2.0 (GraphPad Software, San Diego, CA, USA). The Shapiro-Wilk test assessed the normality of data distribution, while the modified Levene’s test checked the homoscedasticity of the data sets. Comparisons involving more than two groups were performed using one-way analysis of variance (ANOVA). Post-hoc pairwise comparisons were carried out using the Tukey test. For all analyses, statistical significance was set at α = 0.05.

## Results

### Bacterial biofilm removal efficacy

**Fig. 4:**
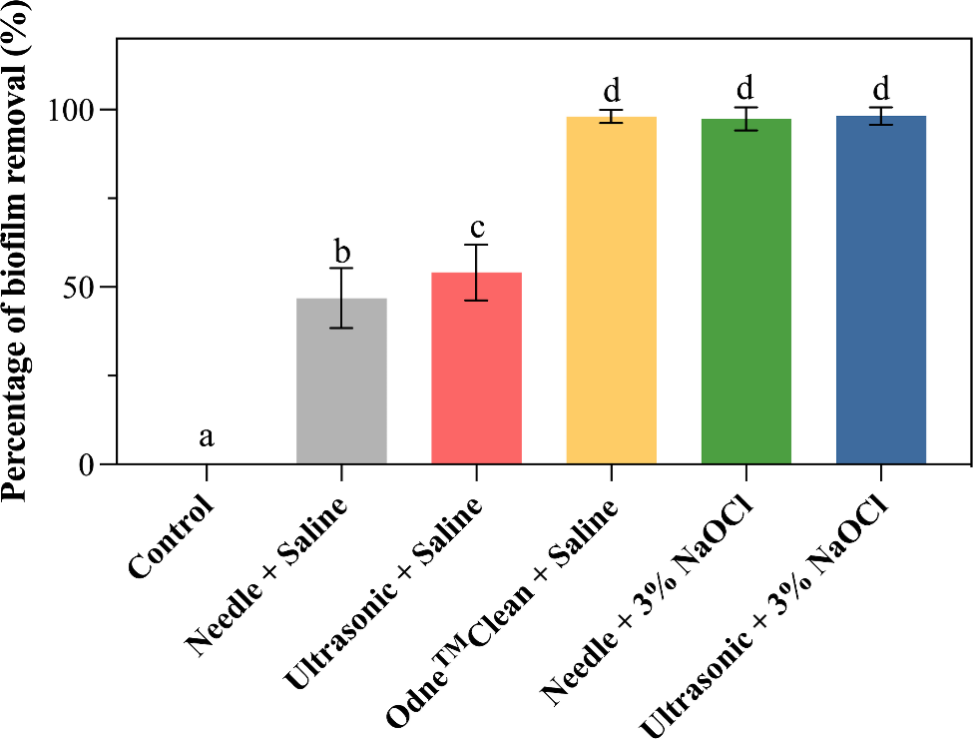
Bar chart showing the percentage of bacterial biofilms removal in control group, Needle + Physiological Saline group, Ultrasonic + Physiological Saline group, Odne^TM^Clean + Physiological Saline group, Needle + 3% NaOCl group, and Ultrasonic + 5% NaOCl group. Different lowercase letters indicate a significant difference between groups (*P* < 0.05)

**Fig. 5:**
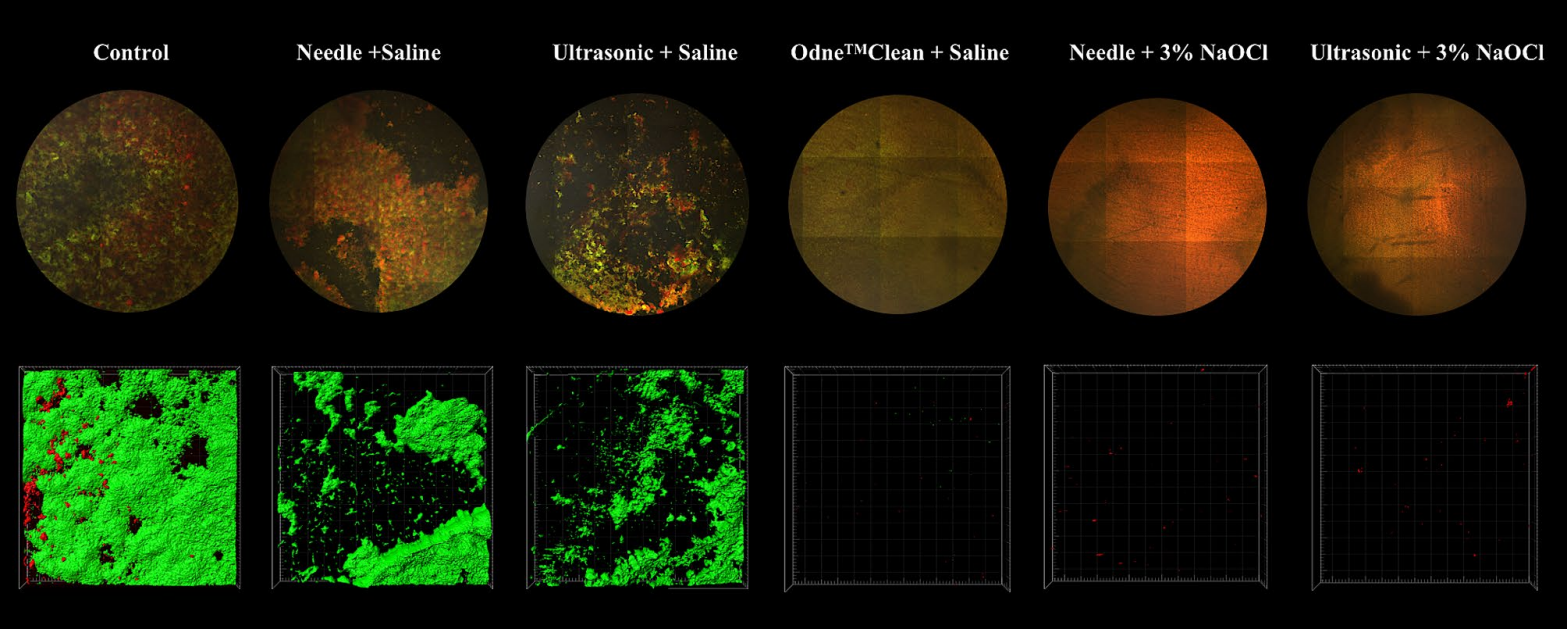
Representative confocal laser scanning microscopy images (upper row: map view) and three-dimensional reconstructions (lower row) in control group, Needle + Physiological Saline group, Ultrasonic + Physiological Saline group, Odne^TM^Clean + Physiological Saline group, Needle + 3% NaOCl group, and Ultrasonic + 5% NaOCl group

Figure 4 presents the percentage of bacterial biofilm removal in each group. There is a significant difference in the percentage of bacterial biofilm removal among the groups (*P* < 0.05, R^2^: 0.9830). When physiological saline was used as the irrigant solution, Odne^TM^Clean (98% ± 2%) performed significantly better than needle irrigation (47% ± 8%) and ultrasonic activation (54% ± 8%) (*P* < 0.05). Odne^TM^Clean with physiological saline removed bacterial biofilm as effectively as needle irrigation with 3% NaOCl (97% ± 3%) and ultrasonic activation with 3% NaOCl (98% ± 2%) (*P* > 0.05). Figure 5 shows representative CLSM images and three-dimensional reconstructions for each group.

### Smear layer and debris removal efficacy

**Fig. 6:**
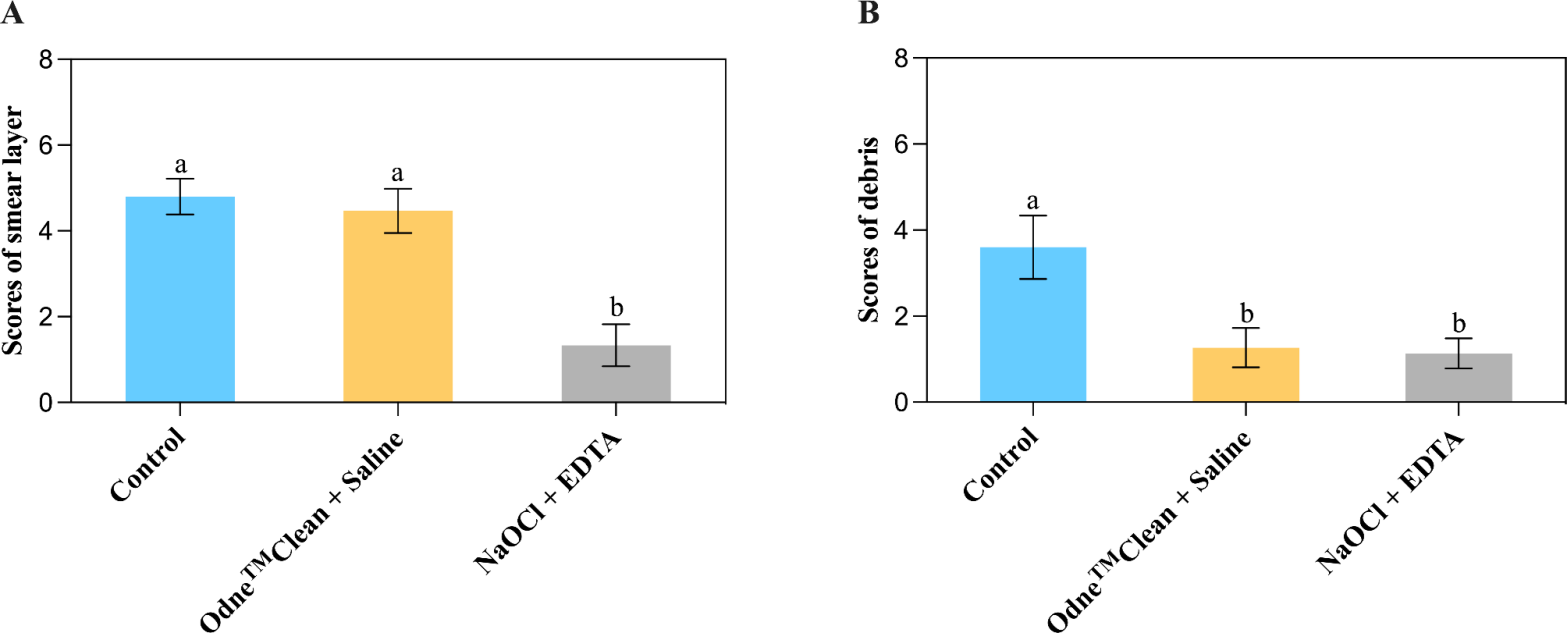
Bar charts showing scanning electron microscopy scores of smear layer (**A**) and debris (**B**) in the control group, Odne^TM^Clean + Physiological Saline group, and 5% NaOCl + 17% EDTA group. Different lowercase letters indicate a significant difference between groups (*P* < 0.05)

There is a significant difference in the percentage of smear layer removal among the groups (*P* < 0.05, R^2^: 0.9206). In the smear layer experiments, 5% NaOCl followed by 17% EDTA (score: 4.47 ± 0.52) removed the smear layer significantly better than Odne^TM^Clean with physiological saline (score: 1.33 ± 0.49) (*P* < 0.05) (Fig. 6A). There was no significant difference between the control group and Odne^TM^Clean with physiological saline (*P* > 0.05) (Fig. 6A).

There is a significant difference in the percentage of debris removal among the groups (*P* < 0.05, R^2^: 0.8248). In the debris experiments, Odne^TM^Clean with physiological saline (score: 1.27 ± 0.46) removed debris as effectively as 5% NaOCl followed by 17% EDTA (score: 1.13 ± 0.35) (*P* > 0.05) (Fig. 6B).

**Fig. 7:**
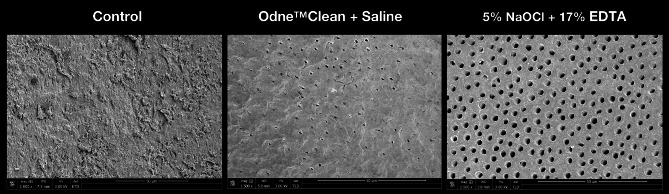
Representative scanning electron microscopy images (scale bar: 50 μm) of smear layer and debris in the control group, Odne^TM^Clean + Physiological Saline group, and 5% NaOCl + 17% EDTA group

The representative SEM image in the control group showed a heavy smear layer covering the dentin disc, with a large amount of debris on the surface (Fig. 7). In the Odne™ Clean + Physiological Saline group, the SEM image revealed a substantial smear layer covering the dentin disc, with a small amount of debris on the surface (Fig. 7). In the 5% NaOCl + 17% EDTA group, the SEM image showed no smear layer covering the dentin disc and a minimal amount of debris (Fig. 7).

## Discussion

Effectively removing biofilm, smear layer, and debris during root canal treatment is crucial for root canal disinfection and periapical healing [2, 12, 15]. The majority of biofilms and debris are removed during root canal instrumentation and irrigation [15, 16]. The removal of the smear layer generated during instrumentation, as well as the remaining biofilm and debris, relies on the final irrigation [15, 16]. Physiological saline is often used as a root canal irrigant between NaOCl and EDTA during the final irrigation [15]. During this time, the activation of the physiological saline solution may help remove the remaining biofilm and debris, a point less addressed in previous studies. Unlike traditional irrigation devices, the Odne^TM^Clean system is designed to activate a physiological saline solution during the final irrigation. This study aimed to compare the biofilm, smear layer, and debris removal efficacy of the Odne^TM^Clean system with double side-vented needle irrigation and ultrasonic activation using a new ex vivo model, employing CLSM and SEM for analysis.

The removal of biofilm and debris from the surface of the root canal wall primarily relies on the dissolution of the biofilm by irrigants such as NaOCl [12, 13], and the hydrodynamic action of the irrigant produced by various irrigation techniques or devices to detach the biofilm from the root canal wall [14]. In the present study, the Odne^TM^Clean system with physiological saline removed the majority of biofilm (98%) from the dentin surface, which was significantly better than needle irrigation (47%) or ultrasonic activation with physiological saline (54%). Therefore, the null hypothesis of this study, which stated that “there would be no significant differences in the biofilm, smear layer, or debris removal efficacy between Odne^TM^Clean and double side-vented needle irrigation or ultrasonic activation,” was rejected.

Interestingly, Odne^TM^Clean with physiological saline removed biofilms as effectively as needle or ultrasonic irrigation with 3% NaOCl. Unlike NaOCl, physiological saline does not have tissue-dissolving ability [15]. Previous studies have demonstrated the significant antimicrobial efficacy of microbubble cavitation [30, 31]. Therefore, the effective biofilm removal by physiological saline was attributed to the cavitation cloud of imploding microbubbles produced by Odne^TM^Clean, generating powerful shockwaves capable of detaching the biofilm from the root canal wall. Additionally, Odne^TM^Clean with physiological saline effectively removed debris from the dentin surface, as shown in SEM images, which revealed only a small amount of debris remaining compared to the control group. These results demonstrate that Odne^TM^Clean with physiological saline can effectively remove biofilms and debris from the dentin surface. In endodontics, physiological saline-based systems such as Odne^TM^Clean offer potential clinical benefits, such as minimizing patient discomfort and reducing cytotoxicity. These systems are biocompatible and non-toxic, making them a safer alternative to traditional irrigants like sodium hypochlorite, which may cause irritation or damage to periapical tissues when extruded beyond the root canal. The use of physiological saline can enhance patient comfort during treatment while still supporting effective cleaning when combined with advanced activation techniques like hydrodynamic cavitation.

The removal of the smear layer primarily relies on the synergistic action of NaOCl and calcium chelating agents such as EDTA: NaOCl removes the organic components of the smear layer, while EDTA removes the inorganic components [12, 15]. In the smear layer experiments, 5% NaOCl followed by 17% EDTA effectively removed the smear layer, as evidenced by SEM images showing no smear layer covering the dentin surface and more than 90% of dentinal tubules open. In contrast, Odne^TM^Clean with physiological saline was not capable of removing the smear layer from the dentin surface, showing no significant difference compared to the control group. This may be because physiological saline is unable to chemically remove the organic components of the smear layer or dissolve its inorganic components [12, 15]. Additionally, the activation of physiological saline by Odne^TM^Clean may not generate sufficient wall shear forces to mechanically remove the smear layer from the root canal walls.

A new ex vivomodel was designed and fabricated in this study. This model effectively simulates the morphology of tooth roots, providing a clinically relevant environment for testing various root canal irrigation techniques. It also enables the standardization of the location of dentin discs, facilitating fair and quantitative comparisons of the biofilm removal efficiency of different irrigation techniques. Moreover, this reproducible model can significantly reduce costs and the consumption of extracted teeth while increasing sample sizes. Finally, dentin discs were bonded to the inner walls of the artificial root with super glue before irrigation and were non-invasively removed with pliers afterward. This method prevents any impact on the biofilm, smear layer, and debris on the dentin discs during handling, thereby ensuring more reliable research results. However, this ex vivo model has a limitation: the volume of the artificial root socket is significantly larger than that of a natural root canal, allowing it to hold a greater amount of irrigant. Future studies should aim to refine the model to more closely replicate the morphology and volume of natural root canals, thereby providing a more clinically relevant platform.

In this study, 3-week-old bacterial biofilms were cultured and used, as they are considered mature for evaluating the biofilm removal efficacy of different irrigation methods. Shen et al. [32] demonstrated that multispecies biofilms cultured from plaque bacteria on collagen-coated hydroxyapatite discs in brain-heart infusion broth exhibited significant growth during the initial weeks. Biofilm thickness increased from 57 μm at 2 days to 155 μm at 3 weeks. After 3 weeks, under nutrient-limiting conditions, the biofilm thickness stabilized, ranging from 190 μm at 6 weeks to 201 μm at 12 weeks. These findings indicate that biofilms reach structural maturity at 3 weeks, as evidenced by the stabilization of thickness. Accordingly, 3-week-old biofilms were selected for this study.

Dentin disinfection is essential for successful root canal treatment as bacteria can colonize deep within the dentin [33, 34]. The interaction of endodontic disinfecting agents with dentin also affects the efficacy of the irrigants [33, 34]. Numerous studies have evaluated the effectiveness of various irrigation techniques in activating solutions like NaOCl for dentin disinfection [17, 35–37]. Although physiological saline does not have antimicrobial properties, further research is needed to determine whether the microbubble cavitation produced by the Odne^TM^Clean system can effectively eliminate bacteria within the dentinal tubules. Furthermore, although the manufacturer does not recommend combining NaOCl with the Odne^TM^Clean system to avoid potential apical extrusion and associated toxicity or damage to the periapical tissue, it would be beneficial for future in vitro studies to evaluate the tissue dissolution and antibacterial efficacy of Odne^TM^Clean activation with NaOCl. Additionally, once extensive laboratory studies have validated the in vitro performance of Odne^TM^Clean, high-quality, long-term randomized clinical trials will be necessary to confirm its in vivo efficacy.

In conclusion, when physiological saline was used as the irrigant solution, Odne^TM^Clean performed significantly better than needle irrigation and ultrasonic activation. Odne^TM^Clean with physiological saline removed bacterial biofilm as effectively as needle irrigation with 3% NaOCl and ultrasonic activation with 3% NaOCl. However, 5% NaOCl followed by 17% EDTA removed the smear layer significantly better than Odne^TM^Clean with physiological saline, which was not effective at removing the smear layer. Additionally, Odne^TM^Clean with physiological saline removed debris as effectively as 5% NaOCl followed by 17% EDTA. Within the limitations of this study, during the final irrigation phase, using Odne^TM^Clean with physiological saline between NaOCl and EDTA irrigation may enhance the cleaning efficacy of the root canal. Future research is needed to evaluate the to evaluate the tissue dissolution and antibacterial efficacy of Odne^TM^Clean activation with NaOCl.

## Electronic supplementary material

Below is the link to the electronic supplementary material.

Supplementary Material 1

## Data Availability

The corresponding author can provide the datasets used and/or analyzed during the current study upon reasonable request.
