# Supplementary figures and images for: Evaluation of bacterial biofilm, smear layer, and debris removal efficacy of a hydro-dynamic cavitation system with physiological saline using a new ex vivo model: a CLSM and SEM study
Source: BMC Oral Health. 2025 Jan 18;25:95. doi: 10.1186/s12903-025-05463-y (PMC11742200; doi:10.1186/s12903-025-05463-y)

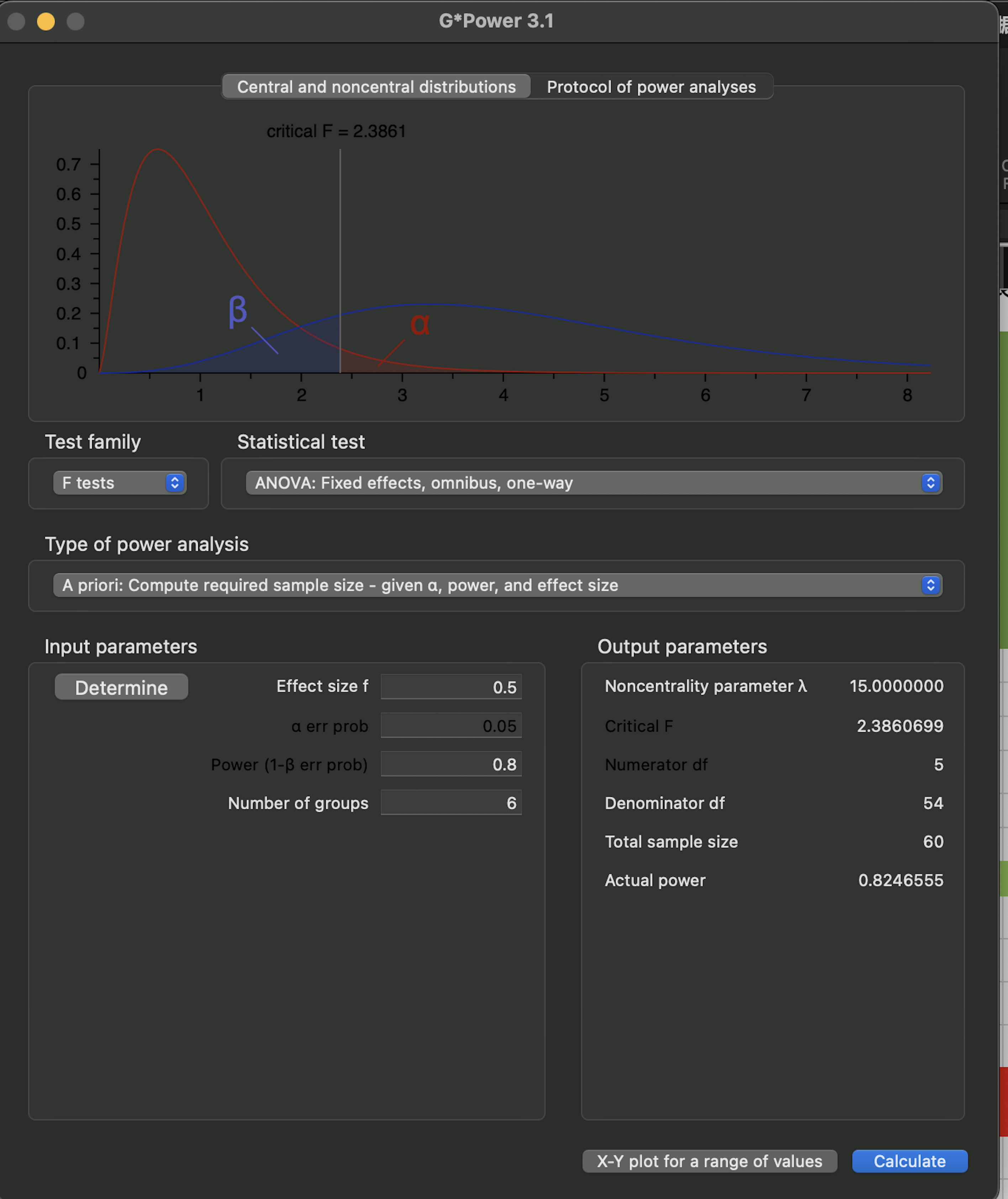

Supplement: Supplementary file 1 — Supplementary Material 1 [file 12903_2025_5463_MOESM1_ESM.png]
